# High overlap of zoonotic helminths between wild mammalian predators and rural dogs – an emerging One Health concern?

**DOI:** 10.1017/S0031182022001032

**Published:** 2022-10

**Authors:** Ants Tull, Harri Valdmann, Egle Tammeleht, Triin Kaasiku, Riinu Rannap, Urmas Saarma

**Affiliations:** Department of Zoology, Institute of Ecology and Earth Sciences, University of Tartu, J. Liivi 2, 50409 Tartu, Estonia

**Keywords:** *Canis aureus*, *Canis familiaris*, *Eucoleus*, genetic identification, Taeniidae, *Toxocara canis*, *Toxocara cati*, *Trichuris*, *Uncinaria stenocephala*, *Vulpes vulpes*, zoonotic parasites

## Abstract

The concept of One Health emphasizes the interdependence of human, animal and environmental health and is of growing significance, in part owing to the problems related to emerging infectious diseases of wildlife origin. Wild mammalian predators are a potential risk factor for transmission of zoonotic pathogens to domesticated animals and humans. This is especially relevant in rural areas, where transmission of zoonotic pathogens can occur particularly efficiently when free-ranging dogs are present. The main aim of this study was to determine helminth infections among wild mammalian predators and evaluate the overlap between helminth faunas of wild mammals and dogs. Scat samples of predators were collected in coastal areas of Western Estonia and genetic methodology applied for the correct identification of predator species from their scat. Parasitic helminths of mammalian predators in the scat samples were analysed and compared with dog data from a previous study. High helminth prevalence (~90%) was found in dominant predator species in the area, namely the red fox (*Vulpes vulpes*) and golden jackal (*Canis aureus*). Moreover, the helminth fauna of both wild species, including potentially zoonotic helminths, overlapped largely with that of rural dogs in the same area. The results, together with the ones from earlier parasitological studies among humans in Estonia, emphasize the potential risk of pathogen transmission from wild mammalian predators to dogs and from dogs to humans, making parasitic diseases of wildlife a One Health concern.

## Graphical abstract

**Figure:**
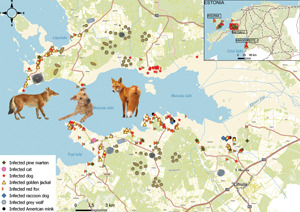

## Introduction

Humans are altering ecosystems on a scale never seen before, which has led to problems from high environmental pollution and biodiversity loss to outbreaks of infectious diseases (McMahon *et al*., 2018; Keys *et al*., 2019). The continuous fragmentation of natural habitats has increased contacts of wildlife with domesticated animals and humans, leading to the risk of pathogen concentration and spillover (Deplazes *et al*., 2004; Faust *et al*., 2018) and making parasitic diseases of wildlife a rising One Health concern (Waindok *et al*., 2021; Casulli *et al*., 2022). One Health emphasizes the interdependence of human, animal and environmental health, and emerging infectious diseases of wildlife origin have made the concept of considerable importance (Jenkins *et al*., 2015).

Among the most abundant mammalian carnivores in Europe and Estonia are the red fox (*Vulpes vulpes*) and raccoon dog (*Nyctereutes procyonoides*), and together with the golden jackal (*Canis aureus*), they are displaying opportunistic food habits (Kauhala *et al*., 1998; Baltrunaite, 2002; Soe *et al*., 2017; Lange *et al*., 2021). Their food includes small mammals, insects, reptiles and fish that can act as vectors or paratenic hosts for helminths, playing an important role in transmissions of parasitic helminths of zoonotic potential, including tapeworms such as *Echinococcus* spp., *Taenia* spp., and roundworms like *Trichinella* spp. and *Toxocara* spp. Although a number of zoonotic diseases caused by parasitic helminths have been drastically reduced due to improved hygiene and prophylactic treatment, many parasitic zoonoses remain a considerable health and economic burden due to ineffective control programmes. Mammalian predators such as the red fox, golden jackal, pine marten (*Martes martes*) and raccoon dog are common definitive hosts for many zoonotic helminths in Estonia and many other countries in Europe and beyond. However, these species are known to move often in areas where domestic animals and humans reside (e.g., Plumer *et al*., 2014; Süld *et al*., 2017*a*). Defecating in these areas contaminates the environment with various pathogens, including zoonotic helminths. Studies have found that in Europe, raccoon dogs are infected with a minimum of 32 helminth species of which 19 are zoonotic (Laurimaa *et al*., 2016*a*). It is also known that red foxes and golden jackals are hosts for many helminth parasites, most of them infecting also domestic dogs and cats (e.g., Otranto *et al*., 2015; Gherman and Mihalca, 2017; Karamon *et al*., 2018). Moreover, foxes have been colonizing urban areas in Europe, including Estonia, bringing zoonotic pathogens to the vicinity of humans and their pets (Deplazes *et al*., 2004; Laurimaa *et al*., 2015*a*).

Red fox and raccoon dog are among the most widely distributed canid species in Estonia and act as vectors of multiple zoonotic diseases, including alveolar echinococcosis, trichinellosis and sarcoptic mange (Süld *et al*., 2014, 2017*b*; Laurimaa *et al*., 2015*b*, 2016*a*, 2016*b*; Marcinkutė *et al*., 2015; Kärssin *et al*., 2017). In Estonia, a total of 17 endoparasite taxa has been found in red foxes, 10 of them with zoonotic importance (Laurimaa *et al*., 2016*b*), whereas for the raccoon dog the corresponding numbers were 17 and 9 (Laurimaa *et al*., 2016*a*).

The golden jackal was included into the list of mammal species in Estonia nearly a decade ago and ever since the population has grown, mainly in Western Estonia, sharing overlapping territories with other wild and domestic animals (e.g., red foxes, raccoon dogs, dogs), and also humans. It is therefore important to assess which helminth infections the golden jackal as well as other canids transmit, especially those of zoonotic potential.

Apart from wild mammals, dogs can also be an important source of helminth infection, both in rural and urban areas. Recent investigations have found that about 10% of scats of urban dogs in Estonia were infected with the eggs of helminths (Tull *et al*., 2020), whereas in rural areas the infection rate was significantly higher, reaching up to 87% (Tull *et al*., 2022). A zoonotic tapeworm parasite *Echinococcus granulosus* has also been recorded in dogs in Estonia (Laurimaa *et al*., 2015*c*).

Estonia, especially its western part, provides the best opportunities to study the environmental contamination with the eggs of helminth parasites in a complex manner, analysing the role of mammalian predators, including red foxes and golden jackals, but also rural dogs. This part of the country hosts the largest population of golden jackals in Estonia (in other areas of the country the species is scarce) and, in addition, the data for rural dog helminths from this area has been obtained in a previous study (Tull *et al*., 2022).

The aims were to: (1) determine helminth infections among wild mammalian predators; (2) evaluate the overlap between helminth faunas of different wild and domestic canids such as the red fox, golden jackal and dog; (3) assess the effect of diet on the risk of infection with helminths.

## Materials and methods

### Study area and samples

**Fig. 1.**
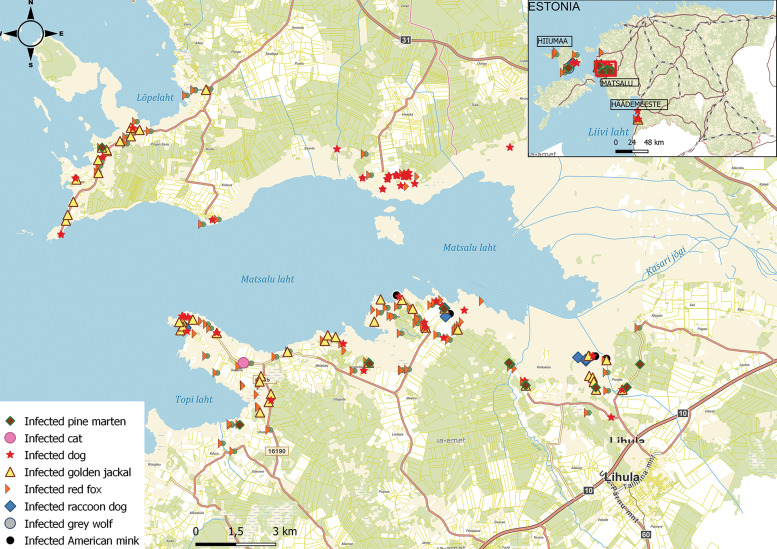
Collection sites of predator scats infected with parasitic helminths (*n* = 287) in western part of Estonia. Samples are mainly from the Matsalu National Park (enlarged), but also from Hiiumaa and Häädemeeste. Dog data are from Tull *et al*. (2022).

A non-probabilistic sampling was used for collecting scats of mammalian predators in rural areas of Western Estonia, namely in Matsalu National Park, Häädemeeste and Hiiumaa in April–June 2019 (Fig. 1). Each sample was placed into a separate plastic bag and tagged with unique ID, including global positioning system coordinates. To inactivate eggs of zoonotic parasites, for example *Echinococcus multilocularis* and *E. granulosus s.l.*, which are endemic in Estonia (Moks *et al*., 2006, 2008; Laurimaa *et al*., 2015*a*, 2015*b*), samples were kept at −80°C for a minimum of 7 days. Data for dog samples (*n* = 84), that are described in detail in Tull *et al*. (2022) and are from the same study area, were also included to provide a comparison with other mammalian predators.

### Molecular identification of mammalian predators

Scats of different canid species are sometimes difficult to distinguish and to avoid mixing the data of various species, a genetic analysis was conducted for correct identification of mammalian species. Genomic DNA was isolated from scats using QIAamp Fast DNA Stool Mini Kit (Qiagen, Hilden, Germany) according to manufacturer's protocol. A hypervariable fragment of the mitochondrial DNA (mtDNA) control region that enables distinguishing wolves and dogs in Estonia, was PCR-amplified and sequenced as described in Plumer *et al*. (2018). This mtDNA fragment allows also distinguishing other predators (Tull *et al*., 2022). In brief, a 351 base-pair (bp) fragment of the mtDNA control region was PCR-amplified using 0.25 μM of primers Canis1F and Canis3R. The reaction mixture (20 *μ*l in total), contained 2 *μ*L of DNA, 1 × Phusion HF buffer, 0.4 mm dNTP and 0.4 U Phusion HS II polymerase (Thermo Fisher Scientific, Waltham, USA). The following PCR cycling parameters were used: 30 s at 98°C, then 10 cycles: 10 s at 98°C, 30 s at 68°C (with touchdown of −0.8°C per cycle), 45 s at 72°C; then 35 cycles: 10 s at 98°C, 30 s at 60°C, 45 s at 72°C and finally 2 min at 72°C. PCR products were purified with 1 U of both FastAP and ExoI (Thermo Fisher Scientific). Purified PCR products were sent for sequencing to the core laboratory of the Institute of Genomics at the University of Tartu.

Sequences of both DNA chains were aligned with CodonCode Aligner v.9.0.2 (CodonCode Corp; Centerville, MA, USA) to produce consensus sequences and corrected using BioEdit v.7.2.5 (Hall, 1999). The final alignment was 245 bp and the dataset was further aligned with homologous wolf (Hindrikson *et al*., 2012; Plumer *et al*., 2018), red fox and golden jackal (Tull *et al*., 2022) sequences from Estonia.

### Molecular identification of food objects

For the identification of birds, mammals, reptiles and fish, a 303 bp fragment of mtDNA *cox1* gene was PCR-amplified with primers AVS2F and AVS3R as described in Tull *et al*. (2022). PCR reactions were carried out in a total volume of 20 *μ*L with 1× Phusion HF Buffer (Thermo Fisher Scientific), 0.2 mm dNTP, 0.25 *μ*m of each primer and 0.4 U Phusion Hot Start II DNA Polymerase and 2 *μ*L of purified DNA. The PCR mixture was initially denatured at 98°C for 30 s, followed by 10 touchdown cycles for 10 s at 98°C, 20 s at 60°C (reducing the temperature 1°C per cycle) and 30 s at 72°C, followed by 30 cycles of 10 s at 98°C, 20 s at 50°C and 30 s at 72°C. In case the PCR was negative due to highly degraded DNA, we performed a second analysis by PCR-amplifying a shorter, 183 bp fragment of mtDNA 12S rRNA gene, using primers Ave12F and Ave12R, described in Oja *et al*. (2017). PCR products were checked using 2% 1×TAE gel-electrophoresis and visualized under UV radiation using ethidium bromide.

PCR products were purified, sequenced and nucleotide BLAST (https://blast.ncbi.nlm.nih.gov/Blast.cgi) was used to identify various taxa, such as reptiles, fish and birds.

### Morphological analysis of food objects

The analysis was done as described in Valdmann and Saarma (2020). Shortly, fecal samples were processed according to standard laboratory procedures (Reynolds and Aebischer, 1991). Non-mammal remains (e.g., birds) recovered in predator scats were identified in comparison with reference materials. Mammal remains were identified by examining the cuticular pattern and the medulla of the hairs using reference manuals (Teerink, 1991; Tóth, 2017) and hairs collected from hunted animals.

### Parasite identification and prevalence

Helminth occurrence was determined using the concentration flotation technique (NaCl and glucose solution, specific gravity 1.2–1.3 g cm^−3^) (Roepstorff and Nansen, 1998), followed by helminth egg counting in McMaster chambers until 100 eggs per parasite taxa. Identification of helminth taxa was based on morphological characteristics (Bowman, 2013). Most of the parasite taxa were identified at the genus level, except *Toxocara canis*, *Toxascaris leonina* and *Uncinaria stenocephala*. Although we attempted genetic identification of species among the isolated eggs of Taeniidae, it was not successful, possibly due to degradation of DNA.

Helminth prevalence was defined as the number of predator host infected with particular helminth taxa divided by the number of hosts examined for that helminth taxa. The relative infection intensity was determined as the count of eggs up to 100 per taxa in a sample.

### Spatial analysis

Maps were created using the QGIS (v3.24) to visualize the geographical locations of predator scats and to calculate the average distance between private houses (henceforth privates) and all the collected scat samples (QGIS Development Team, 2022). This buffer distance around scat samples was considered as the average free-ranging area of mammalian predators from privates (Fig. 1). Another buffer was generated by joining together the first buffer with the infected scats layer situated inside the buffer zone to count privates in the potential hazard zone. The map layers originated from public WMS services (Estonian Land Board, 2022).

### Statistical analysis

The dependent variables consisted of (co)infection prevalence and infection intensity. The independent variable consisted of food items (data not given; a separate manuscript on the diet of predators is in preparation). Food objects were divided into 5 categorical variables: game (mammals), bird, dog food, plant material and rodent.

Since multiple testing was performed between (co)infection and different food groups, it is possible to obtain false-positive results (Type I error) in a set of tests. The Holm–Bonferroni method, which is more powerful compared to Bonferroni procedure, was applied to prevent Type I error rates when performing multiple tests (Aickin and Gensler, 1996).

Proportions were compared with SAS Studio (3.8) software using chi-squared tests of independence (PROC FREQ) to determine independent variables associated with overall (co)infection and single taxa prevalence (SAS Institute Inc, 2022). If 1 or more cells in the 2 × 2 contingency tables had expected values of less than 5, Fisher's exact test was used. Generalized linear models (GLM) package ‘glmmTMB’ (Brooks *et al*., 2017; R Core Team, 2022) was used to evaluate consumption of various food objects associated with individual and overall helminth prevalence and with the overall coinfection prevalence. It was also estimated how red fox and golden jackal diet associates with prevalence (binomial error distribution) and intensity (negative binomial error distribution) of individual helminth taxa. Models were compared using the Akaike information criterion corrected for small samples (AICc) (Burnham *et al*., 2002). Package ‘MuMIn’ (Barton, 2019) was used for conducting model selection and model averaging. Only models with the highest Akaike weight *wi*(AIC) (ΔAICc < 2) were described as the model with the highest Akaike weights provide a continuous measure of strength of evidence. It is especially important to assess the weight of evidence in favour of the best model when a binary decision is made and the other candidate models (with higher AIC values) are simply discarded (Wagenmakers and Farrell, 2004). Furthermore, the weights (*wi*) of the same factors presented in 1 model set were summed for calculating the relative variable importance (RVI).

The non-parametric Mann–Whitney U test was used to compare the mean parasite richness between 3 larger canids (red fox, golden jackal, dog). All presented results of statistical tests had *P* value < 0.05.

To measure the overlap of helminths between 2 host species, the Pianka's index (Pianka, 1973) was calculated using the following formula:
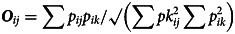
where *p_ij_* and *p_ik_* are proportions of parasite taxa in the hosts *j* and *k* respectively, expressed as percentage.

## Results

**Fig. 2.**
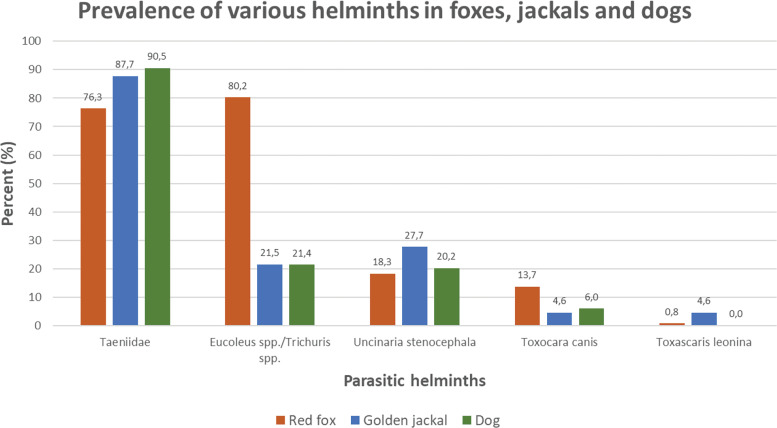
Prevalence of different parasitic helminths in scats of the 3 most abundant mammalian predator species: red fox (*n* = 131), golden jackal (*n* = 65) and dog (*n* = 84).

Based on the 624 scat samples collected from the coastal areas in Western Estonia, genetic analysis identified predator species for 315 samples. Among these, the analysis based on morphological examination of parasite ova revealed that 91.1% (*n* = 287) were infected (Table 1; Supplementary Material Fig. S1). Out of the infected scats, 38.3% (*n* = 110) harboured a single helminth taxon and 61.7% (*n* = 177) were coinfected with 2 or more helminth taxa (Supplementary Material Fig. S2). Majority of predator scats harboured eggs of Taeniidae (84.1%), followed by *Eucoleus* spp./*Trichuris* spp. (50.2%), *U. stenocephala* (20.3%), *T. canis* (8.9%), *T. leonina* (1.3%) (Table 1; Fig. 2). Eggs of *Toxocara cati* were found only in a single cat sample (0.3%) (Table 1).

**Table 1.** Prevalence of zoonotic helminths in scats of different mammalian predators (*n* = 315) of which 287 were infected

	Fox (*n* = 131)	Jackal (*n* = 65)	Dog (*n* = 84)	Pine marten (*n* = 19)	Raccoon dog (*n* = 7)	Mink (*n* = 5)	Wolf (*n* = 2)	Cat (*n* = 1)	Otter (*n* = 1)	Total (*n* = 315)
Helminth positive	92.4 (121)	90.8 (59)	86.9 (73)	100 (19)	100 (7)	100 (5)	50 (1)	100 (1)	100 (1)	91.1 (287)
Helminth negative	7.6 (10)	9.2 (6)	13.1 (11)	0	0	0	50 (1)	0	0	8.9 (28)
Taeniidae^a^	76.3 (100)	87.7 (57)	90.5 (76)	100 (19)	71.4 (5)	100 (5)	50 (1)	100 (1)	100 (1)	84.1 (265)
*Euc/Tri*	80.2 (105)	21.5 (14)	21.4 (18)	63.2 (12)	71.4 (5)	40 (2)	50 (1)	100 (1)	0	50.2 (158)
*Uncinaria stenocephala*	18.3 (24)	27.7 (18)	20.2 (17)	0	42.9 (3)	20 (1)	50 (1)	0	0	20.3 (64)
*Toxocara canis*	13.7 (18)	4.6 (3)	6.0 (5)	5.3 (1)	0	20 (1)	0	0	0	8.9 (28)
*Toxascaris leonina*	0.8 (1)	4.6 (3)	0	0	0	0	0	0	0	1.3 (4)
*Toxocara cati*	0	0	0	0	0	0	0	100 (1)	0	0.3 (1)

Dog data are from Tull *et al*. (2022). The first number is per cent (%) and the number of samples (*n*) is in parentheses. Euc/Tri: *Eucoleus* spp.*/Trichuris* spp.

a Note that all helminth taxa listed in the table are of zoonotic potential. However, when some of the taeniids (e.g., *Echinococcus* spp., *Taenia hydatigena, T. crassiceps*) are zoonotic and a serious threat to human health, there are some that are not zoonotic (e.g., *T. pisiformis, T. cervi*).

Vast majority of red fox scat samples were infected (92.4%; Table 1; Supplementary Material Fig. S1). The most common infection was with *Eucoleus* spp./*Trichuris* spp. (80.2%), followed by Taeniidae (76.3%) and *U. stenocephala* (18.3%) (Table 1; Fig. 2). Among dog scats, 86.9% had helminth ova and the most common taxa were Taeniidae (90.5%), followed by *Eucoleus* spp./*Trichuris* spp. (21.4%) and *U. stenocephala* (20.2%). The golden jackal samples had comparably high proportion of infected scats (90.8%) with the red fox and dog. Golden jackal scats contained most frequently eggs of Taeniidae (87.7%), followed by *U. stenocephala* (27.7%) and *Eucoleus* spp./*Trichuris spp*. (21.5%). The non-parametric Mann–Whitney U test showed that red foxes had significantly higher parasite richness than dogs (*P* < 0.0001) and also higher than golden jackals (*P* < 0.0001). There was no significant difference between dogs and golden jackals.

Among all coinfected scats (*n* = 177), di-infections were the most frequent (75.1%), followed by tri-infections (22.6%) (Supplementary Material Fig. S3). Di-infection with Taeniidae and *Eucoleus* spp./*Trichuris* spp. dominated (52.5%), followed by di-infection with Taeniidae and *U. stenocephala* (14.1%), and tri-infection of Taeniidae, *Eucoleus* spp./*Trichuris* spp. and *U. stenocephala* (14.1%) (Fig. S4).

**Fig. 3.**
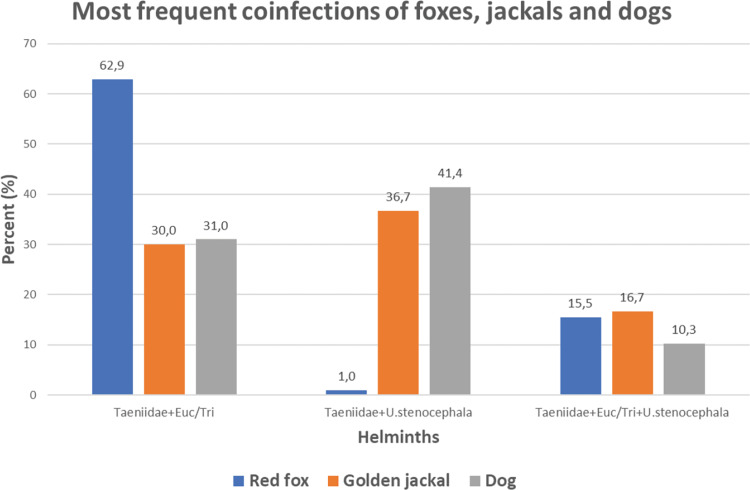
Three most frequent helminth coinfections among coinfected scats of red foxes (*n* = 97), golden jackals (*n* = 30) and rural dogs (*n* = 29). Euc/Tri – *Eucoleus* spp./*Trichuris* spp.

Red fox had the highest coinfection rate (80.2%) (Supplementary Material Fig. S2) of which the most prominent was coinfection with Taeniidae and *Eucoleus* spp./*Trichuris* spp. (62.9%) (Table 2; Fig. 3). The golden jackal had significantly lower coinfection rate (50.8%) of which the most frequent was coinfection with Taeniidae and *U. stenocephala* (36.7%). Among the scats of rural dogs, 39.7% harboured more than 1 helminth taxon. The most prevalent coinfection in dogs occurred between Taeniidae and *U. stenocephala* (41.4%).

**Table 2.** Helminth coinfections among different mammalian predators (*n* = 177)

	Fox	Jackal	Dog	Pine marten	Raccoon dog	Mink	Wolf	Cat	Total
Taeniidae + Euc/Tri	62.9 (61)	30 (9)	31.0 (9)	91.7 (11)	40 (2)	50 (1)	100 (1)		53.1 (94)
Taeniidae + Euc/Tri + U.ste	15.5 (15)	16.7 (5)	10.3 (3)		20 (1)				13.6 (24)
Taeniidae + Euc/Tri + T.can	7.2 (7)		10.3 (3)	8.3 (1)					6.2 (11)
Euc/Tri + T.can	6.2 (6)								3.4 (6)
Euc/Tri + U.ste	2.1 (2)				20 (1)	50 (1)			2.3 (4)
Taeniidae + Euc/Tri + T.can + U.ste	2.1 (2)		6.9 (2)						2.3 (4)
Taeniidae + Euc/Tri + T.can + T.leo + U.ste	1.0 (1)								0.6 (1)
Taeniidae + T.can	1.0 (1)	6.7 (2)							1.7 (3)
Taeniidae + U.ste	1.0 (1)	36.7 (11)	41.4 (12)		20 (1)				14.1 (25)
Taeniidae + U.ste + T.can	1.0 (1)								0.6 (1)
Taeniidae + U.ste + T.leo		6.7 (2)							1.1 (2)
Taeniidae + T.can + T.leo		3.3 (1)							0.6 (1)
Taeniidae + Euc/Tri + T.cat								100 (1)	0.6 (1)
Total	80.2 (97)	50.8 (30)	39.7 (29)	63.2 (12)	71.4 (5)	66.7 (2)	100 (1)	100 (1)	(177)

The first number is per cent (%) and the number of samples (*n*) is in parentheses. Euc/Tri: *Eucoleus* spp.*/Trichuris* spp; T.can: *Toxocara canis*; T.cat: *Toxocara cati*; T.leo: *Toxascaris leonina*; U.ste: *Uncinaria stenocephala*.

The best coinfection model (*wi* = 0.22) showed that predators preying on rodents had significantly higher (2.5×) odds to be coinfected with multiple helminth taxa than predators who had not preyed on rodents (*β*_RODENT_ = 0.9, s.e. = 0.3, *P* < 0.0002) (Supplementary Material Table S1). All other model with the ΔAICc < 2 contained factors ‘rodent’ ‘plant’, ‘fish’ and ‘game’. The RVI indicated very strong effect for the factor ‘rodent’ but weak effect towards factors ‘plant’, ‘fish’ and ‘game’.

The infection prevalence model (*wi* = 0.25) with helminths indicated that predators preying on reptiles had 5.5 times higher odds to be infected with *Eucoleus* spp./*Trichuris* spp. (*β*_REPTILE_ = 1.7, s.e. = 0.7, *P* = 0.006) than predators that did not prey on reptiles. Similarly, preying on rodents increased significantly the odds (3 times) of being infected with *Eucoleus* spp./*Trichuris* spp. (*β*_RODENT_ = 1.1, s.e. = 0.3, *P* = 0.00002) among predators compared to those who had not preyed on rodents. The RVI indicated very strong effect for factors ‘rodent’ and ‘reptile’ but weak effect towards other factors like ‘bird’ and ‘game’ (Supplementary Material Table S2).

The best infection intensity model (*wi* = 0.12) with *Eucoleus* spp./*Trichuris* spp. among predators revealed higher infection intensity for predators preying on game (*β*_GAME_ = 0.6, s.e. = 0.3, *P* = 0.048). The RVI indicated strongest effect for factor ‘game’ but weaker on ‘reptile’ and ‘bird’ (Supplementary Material Table S3).

Surprisingly, the infection prevalence model (*wi* = 0.2) indicated that red foxes had up to 4 times higher odds to be infected with *T. canis*, if they consumed plant material (*β*_PLANT_ = 1.4, s.e. = 0.6, *P* = 0.017). In the same model of sets (ΔAICc < 2), only 2 models included factor ‘rodent’ but without significant results. The RVI indicated moderate effect for factor ‘plant’ but weak effects towards factors ‘bird’, ‘insect’, ‘game’ and ‘rodent’ (Supplementary Material Table S4).

Among golden jackals, there were 2 equally good infection models (ΔAICc < 2). The best infection prevalence model (*wi* = 0.4) with helminths *Eucoleus* spp./*Trichuris* spp. revealed that preying on rodents increases significantly (15.5 times) the odds of being infected with *Eucoleus* spp./*Trichuris* spp. (*β*_RODENT_ = 2.6, s.e. = 0.7, *P* = 0.0002) than not preying on rodents. The RVI indicated very strong effect for factor ‘rodent’ but a weak effect towards factor ‘game’ (Supplementary Material Table S5).

The best infection intensity model (*wi* = 0.56) with helminths *Eucoleus* spp./*Trichuris* spp. revealed 60 times higher infection intensity for golden jackals preying on rodents (*β*_RODENT_ = 4.1, s.e. = 1.3, *P* = 0.0008) and 15.5 times higher infection intensity for golden jackals preying on game (*β*_GAME_ = 2.6, s.e. = 1.2, *P* = 0.03). The RVI indicated very strong effect for factor ‘rodent’ but a weak effect towards factor ‘game’ (Supplementary Material Table S6).

The average distance between infected scat samples and privates was 578 metres. The minimum distance of an infected predator from a private household was 0 m (residing in the middle of a living land) and maximum distance was 2285 m. Moreover, 160 households were in the buffer zone of ⩽578 m in which the average distance of an infected scat to a private reached as far as 59 m (CL 48.2–70.8). There was no statistical significance (*P* < 0.05) between the infected predator groups (dog, red fox and golden jackal) in- (⩽578 m) or outside (0–2285 m) the buffer zones.

The overlap of zoonotic helminth fauna, calculated according to the Pianka's index, was highest between rural dogs and golden jackals (0.99) and somewhat lower for golden jackals and red foxes (0.84), as well as rural dogs and red foxes (0.84). The lowest overlap was determined between urban and rural dogs (0.29), followed by urban dogs and red foxes (0.30), and urban dogs and golden jackal (0.34), (Table 3; Supplementary Material Table S7).

**Table 3.** Helminth overlap between different canids (%)

	Rural dog	Red fox	Golden jackal
Urban dog	29.8	30.7	34.4
Rural dog		84.2	99.6
Red fox			84.1

## Discussion

### Molecular identification of mammalian predators

It is often problematic to morphologically differentiate mammalian predator scats and therefore using genetic analysis for correct identification of host is important to avoid false interpretations (Davison *et al*., 2002; Laurimaa *et al*., 2015*b*; Mumma *et al*., 2016; Valdmann and Saarma, 2020; Tull *et al*., 2022). The applied genetic method allowed to distinguish between various predator species, namely the red fox, dog and golden jackal (as the most abundantly represented species in this study), but also raccoon dog, pine marten, American mink (*Neovison vison*), Eurasian otter (*Lutra lutra*), grey wolf (*Canis lupus*) and cat. However, as it was not possible to discern individuals among mammal predators (DNA degradation was too high in scats to apply microsatellite analysis), it is not possible to say how many individuals were involved. Based on the relatively large study area and the abundance of predators (Kaasiku *et al*., 2022), it can be ruled out that the collected scats belong to only a few individuals. Moreover, the aim was not to analyse the parasite prevalence of different individuals, but the general impact of infected mammalian wildlife contaminating the environment with parasite ova.

### Zoonotic helminths of wild predators and rural dogs as a potential One Health concern

The study revealed a very high occurrence of helminth eggs (91.1%) in scats of mammalian predators in Western Estonia. Moreover, a considerable overlap of zoonotic helminth fauna between jackals and rural dogs (99%), as well as between foxes and rural dogs (84%) was found. This poses a potential risk to human health in rural areas, considering that nearly 160 privates were situated from an infected environmental scat on average up to 59 m. It is remarkable how similar the prevalence of different zoonotic helminths was between rural dogs and golden jackals, but also with red foxes (Fig. 2), whereas the comparison between urban and rural dogs, red foxes and jackals revealed much lower helminth overlap (Table 3). It is important to note that all recorded helminth taxa are potentially zoonotic (Table 1). Most taeniid species in dogs have zoonotic importance (e.g., *Taenia* spp., *Echinococcus* spp., *Dibothriocephalus latus*, *Dipylidium caninum*). Larvae of *U. stenocephala* can penetrate the skin and migrate in the top layer of skin, a condition called cutaneous larva migrans (CDC, 2022. The feline and canine bronchial capillarid *Eucoleus aerophilus* is causing pulmonary capillariasis also in humans and it has been suggested that this zoonotic parasite may be mis- and underdiagnosed in humans (Lalošević *et al*., 2008). Although the prevalence of *T. canis* and *Toxascaris leonina* was relatively low, both can cause toxocariasis in humans (Eslahi *et al*., 2020; Rostami *et al*., 2019). Human infections with *T. leonina* seem to be rare (Beaver and Bowman, 1984; Rausch and Fay, 2011; Hoberg *et al*., 2012).

The high prevalence and overlap with zoonotic helminths of rural dogs with jackals and foxes raises a potential One Health concern in rural areas, since rural dogs provide a direct transmission link of zoonotic helminths from sympatric wildlife predators to dogs and from dogs to humans. The high overlap of parasite faunas among canids in rural areas suggests that rural dogs share roaming territories and food habits with jackals and foxes. As the current study provides evidence that rural areas are under continuous helminth contamination, relevant healthcare institutions should focus more on rural areas to diagnose helminth infections among companion animals (especially dogs) and in general human population. The potential One Health concern is supported by a previous study of Lassen *et al*. (2016), who tested antibodies against different zoonotic parasites (*T. canis, Ascaris lumbricoides, Echinococcus* spp., *Taenia solium*, *Toxoplasma gondii*, *Trichinella spiralis*) among different human subgroups in Estonia. It was concluded that considerable infection pressure occurs already in childhood and zoonotic parasitic infections are underdiagnosed or underreported in Estonia. The seroprevalence of *T. canis*, a zoonotic helminth recorded also in our study, was 12.1% and the seroprevalence was higher in animal caretakers than in the general population. Similarly, Remm and Remm (2014) found that dog owners had a significantly higher risk of being infected with *Toxocara* spp. Moreover, their study revealed another statistically significant relationship, namely that the prevalence was higher among those who do not wash their own garden fruit and vegetables (13.6%), compared to those who did (6.2%).

The overall infection prevalence models indicated that preying on diverse paratenic or intermediate hosts increases significantly the infection risk with helminths. If mammalian predators preyed on rodents or reptiles, the infection with *Eucoleus* spp./*Trichuris* spp. was 3–5.5 times higher. Helminths such as *Eucoleus* spp. and *Trichuris* spp. are geohelminths, maturing in the soil up to several weeks before becoming infective. Generally, mammalian predators become infected by ingesting *E. aerophilus* or *T. vulpis* eggs from the environment (attached to plant material or distributed in water and soil), but infection may also occur when consuming invertebrates (earthworms), Norway rats (*Rattus norvegicus*) or reptiles (Rataj *et al*., 2011; Rothenburger *et al*., 2014; Traversa *et al*., 2014; Wolf *et al*., 2014). The high burden of helminth ova in scats of wild mammals and dogs, most of them situated near privates, indicates that rural environment poses a potential risk to human health. It is also worth noting that the parasite burden of dogs and cats in rural areas in Estonia is much higher compared to urban areas (Tull *et al*., 2020, 2022).

### Helminth coinfections

The overall coinfection rate of all mammalian predators was 61.7% (Supplementary Material Fig. S2) and the most dominant was the di-infection with Taeniidae and *Eucoleus* spp./*Trichuris* spp. (Supplementary Material Fig. S4). This can be explained by the highest infection rates with these parasite taxa (Table 1) and both taxa originate most likely from small rodents. The most coinfected host was the red fox (80.2%), while jackals and rural dogs had lower coinfection rates, 50.8 and 39.7%, respectively (Table 2; Supplementary Material Fig. S2). Di-infections dominated in foxes, jackals and dogs, followed by tri-infections. The rates of these 2 infection types were surprisingly similar in all 3 major predator species (Supplementary Material Fig. S3), which can be explained by relatively similar food habits. It is worth noting that fox scats were coinfected with up to 5 parasite taxa simultaneously. In a previous study in Estonia, foxes were found with up to 12 different parasite taxa and the highest proportion of foxes had 7 taxa (Laurimaa *et al*., 2016*b*). However, this study was based on analysis of different internal organs (not scats), which can reveal also parasites that cannot be identified in scats. Thus, the absolute infection and coinfection rates in all mammal species in this study are likely to be higher. Nevertheless, analysis of scats is the most direct way to evaluate the environmental contamination by parasite ova. Moreover, this approach is non-invasive.

A relatively high similarity of helminth coinfections in jackals and dogs was found: di-infections with Taeniidae + *U. stenocephala* and Taeniidae + *Eucoleus* spp./*Trichuris* spp., but also tri-infection with Taeniidae + *Eucoleus* spp./*Trichuris* spp. + *U. stenocephala* (Table 2; Fig. 3). This is most likely due to sharing overlapping feeding areas and prey objects, facilitating analogous endoparasite transmission. Lanszki *et al*. (2006) have suggested analogous scenario for red foxes and jackals. However, there are also marked differences in coinfections (Table 2; Fig. 3). Foxes have about twice as high coinfection rate with Taeniidae and *Eucoleus/Trichuris* compared to jackals and dogs. Moreover, when foxes are virtually lacking di-infection with Taeniidae and *U. stenocephala*, it was found in nearly 40% of scats of jackals and dogs. This can be explained by high rate of simultaneous coinfection with Taeniidae and *Eucoleus/Trichuris* (Table 2; Fig. 3) by eating small rodents as the host for both helminth taxa. Small rodents are one of the most frequent prey of foxes. The high coinfection prevalence with zoonotic parasites and overlap in dogs, foxes and jackals is worryingly high, representing high risk for human health in rural areas.

### Helminths of the red fox

A very high proportion of fox scats analysed were infected with parasitic helminths (92.4%; Table 1). The main helminth taxa found were *Eucoleus* spp./*Trichuris* spp., Taeniidae, *U. stenocephala* and *T. canis*. Somewhat similar results have been reported by Saeed *et al*. (2006) in Denmark and Bružinskaitė-Schmidhalter *et al*. (2012) in Lithuania. In a previous study of fox parasitic helminths in Estonia that was based on examination of internal organs, 17 helminth taxa were found, including 10 zoonotic and the helminth prevalence was 100%, i.e., all analysed individuals were infected (Laurimaa *et al*., 2016*b*). The highest infection rate was observed for trematode *Alaria alata* (90.7%), followed by nematodes *Eucoleus aerophilus* (87.6%) and *U. stenocephala* (84.3%), all zoonotic helminths. The prevalence of zoonotic taeniid *Echinococcus multilocularis* (that causes alveolar echinococcosis in humans) was 31.5%. Laurimaa *et al*. (2016*b*) found also tapeworms with zoonotic potential not registered in this study, namely *Mesocestoides* spp., *Dibothriocephalus* sp. and *E. multilocularis*. In the current study it was not possible to genetically distinguish between species of Taeniidae, most likely due to DNA degradation of ova by UV radiation in spring season. Since coastal areas offer a wide variety of intermediate or reservoir hosts that are essential for transmission of the above-mentioned taeniids, it is important to determine tapeworm species in further studies to estimate the zoonotic potential and proportions of these helminths in foxes, but also in other potential definitive hosts such as jackals, dogs and raccoon dogs.

The prevalence of *T. canis* in fox scats was rather low (6.0%; Table 1) compared to other countries, for example Germany (43.8%; Waindok *et al*., 2021), Lithuania (40.5%; Bružinskaitė-Schmidhalter *et al*., 2012) or Denmark (59.4%; Saeed *et al*., 2006). The previous fox study in Estonia by Laurimaa *et al*. (2016*b*) found *T. canis* in 29.6% of samples. The difference could be due to lower environmental contamination of *T. canis* in the coastal area. However, there could be another explanation. In Estonia, the food of red foxes includes rodents, mainly in autumn when the abundance of small rodents is the highest due to ripened crops on the fields. During spring, red foxes switch from rodents to food sources like birds, reptiles, hares and amphibians, but also fish in coastal areas (Kauhala *et al*., 1998; Laurimaa *et al*., 2016*b*; Kaasiku *et al*., 2022). The change in the food composition in spring may also reflect a shift in endoparasite species. For example, the relatively low prevalence of *T. canis* in this study could be a result of switching from rodents to other prey items, whereas the infection prevalence of taeniids and *Eucoleus* spp./*Trichuris* spp. is relatively high due to high fish, reptile and amphibian abundance.

It is worth noting that the infection prevalence model indicated a relation between consumed plant material and infection with *T. canis*, namely the infection with *T. canis* increased when the diet consisted more of plant material. One explanation could be the self-medication with plants to lower the burden of helminths. Laurimaa *et al*. (2016*a*) has also suggested a self-medicating behaviour, but for raccoon dog. They found significantly more consumed plants among raccoon dogs with higher helminth prevalence.

### Helminths of the golden jackal

The first presence of golden jackals in Estonia was reported in 2013 and it has been suggested that the species migrated naturally to Estonia, having the genetic origin in the Caucasian population (Rutkowski *et al*., 2015). As a result of such migration, jackals as a definitive host for a number of helminths can increase transmission risk of parasitic species, including those of zoonotic potential (i.e., *Echinococcus* spp., *T. canis*) (Ilić *et al*., 2016; Lalošević *et al*., 2016; Gherman and Mihalca, 2017). In Estonia, Jõgisalu *et al*. (2019) have found, based on analysis of internal organs, that the most prevalent endoparasites of jackals are *A. alata* and *U. stenocephala.* In the current study, the golden jackals were mostly infected with *Taeniidae* and *U. stenocephala*, while it was lower for *Eucoleus* spp./*Trichuris* spp., *T. canis* and *T. leonina*. The infection prevalence and intensity of helminths is largely impacted by preying on rodents. However, coastal areas provide during the spring season not as many rodents as in autumn. Moreover, in springtime a myriad of intermediate and paratenic hosts are available, including amphibians, reptiles, fish and insects, capable of infecting jackals with a wide diversity of endoparasites. Gherman and Mihalca (2017) have reported a total of 194 parasite species distributed by jackals, a majority of these parasites are shared with dogs or cats. Due to the relatively high infection prevalence with *Taeniidae* and *U. stenocephala*, the importance of golden jackal as a definitive host for zoonotic *Echinococcus* spp., hookworms and *T. canis* should be highlighted because of the hazards to public health, and further investigations should focus more on diagnosing these zoonotic parasites at a species level. This requires relatively fresh scats and a more refined genetic methodology.

### Helminths of other mammal species

The other mammalian predators infected with helminths were the pine marten, raccoon dog, mink, wolf, cat and otter (Table 1). The American mink and raccoon dog are non-native mammal species in Estonia (similar to the golden jackal), capable of transmitting endoparasites. Raccoon dogs were predominantly infected with Taeniidae and *Eucoleus/Trichuris* (both 71.4%). Former studies in Estonia, based on examination of internal organs, have shown that the overall prevalence of cestodes among raccoon dogs was 30.5% (Laurimaa *et al*., 2016*b*), more than 2 times lower compared to the current study, which could be due to various reasons (difference in sampling locations, time, etc.). Regarding Taeniidae, raccoon dogs can act also as a vector for the tapeworm *E. multilocularis* (1.6%; Laurimaa *et al*., 2015*c*), albeit to a much lower extent compared to the red fox (31.5%; Laurimaa *et al*., 2016*a*).

Interestingly, according to our data, mink and pine marten distribute *Toxocara* spp. ova, which may suggest that they can act as definitive hosts for *Toxocara* spp., although Kołodziej-Sobocińska *et al*. (2020) have indicated that American mink serves rather as a paratenic hosts for *T. canis* in the wild. It is possible that eggs of unembryonated *T. canis* were swallowed from the contaminated environment and passed *via* scats again to the environment. Nevertheless, if mink and marten serve as a paratenic host for *T. canis*, this zoonotic parasite can spread to definitive hosts such as red foxes, golden jackals or dogs. In Poland, Borecka *et al*. (2013) reported that pine martens were infected with zoonotic *T. cati* in the Tatra National Park. The single cat sample in this investigation was also infected with *T. cati*, whereas a recent study by Tull *et al*. (2021) found that 45.6% of analysed rural cat samples (among shelter cats) were infected with this parasite.

To conclude, the study revealed the role of wild mammals, especially the red fox and golden jackal, in contaminating rural environment with eggs of zoonotic helminths. The importance of the golden jackal, a new mammal species in Estonia and several other European countries, as a vector of zoonotic helminths and a potential hazard to public health, should be emphasized. Considering that habitats of wild predators such as the red fox and golden jackal overlap to some extent with free-ranging dogs, endoparasite transmission may occur, being potentially also hazardous to humans who interact with infected dogs. Moreover, humans can also get infection from the contaminated environment. Based on the results, it should be recommended that free-ranging of dogs in rural areas is limited as much as possible and that dogs are regularly treated with appropriate anthelmintics. Moreover, further parasitological investigations of jackals and other wild mammals have to be conducted to reveal the full spectrum of zoonotic helminths at a species level. It is worth noting that human infection with zoonotic parasites is likely underdiagnosed and the zoonotic potential of many helminth parasites is therefore not fully revealed. As many parasitic diseases of wildlife are a One Health concern, a coordinated work between healthcare institutions, veterinarians and parasitologists are required to diagnose helminth infections among wild mammals, companion animals and in general human population in Estonia.

## Data Availability

Data is available upon request.

## Supplementary material

For supplementary material accompanying this paper visit https://doi.org/10.1017/S0031182022001032. click here to view supplementary material
