# Supplementary material for: High overlap of zoonotic helminths between wild mammalian predators and rural dogs – an emerging One Health concern?
Source: Parasitology. 2022 Aug 4;149(12):1565–74. doi: 10.1017/S0031182022001032 (PMC11010195; doi:10.1017/S0031182022001032)
Supplement: Supplementary file 1 [file S0031182022001032sup001.docx]

Supplementary material

**High overlap of zoonotic helminths between wild mammalian predators and rural dogs – an emerging One Health concern?**

Ants Tull, Harri Valdmann, Egle Tammeleht, Triin Kaasiku, Riinu Rannap and Urmas Saarma*

Department of Zoology, Institute of Ecology and Earth Sciences, University of Tartu, Juhan Liivi 2,

50409 Tartu, Estonia.

**Author for correspondence:** Urmas Saarma; E-mail: [urmas.saarma@ut.ee](mailto:urmas.saarma@ut.ee)

**Table of contents**

| **Table S1.** Models predicting coinfection prevalence among predators | **p3** |
| --- | --- |
| **Table S2.** Models predicting infection prevalence with *Eucoleus* spp*./Trichuris* spp. among predators | **p4** |
| **Table S3.** Models predicting infection intensity with *Eucoleus* spp./*Trichuris* spp. among predators | **p5** |
| **Table S4.** Models predicting infection prevalence with *Toxocara canis* among red foxes | **p6** |
| **Table S5.** Models predicting infection prevalence with *Eucoleus* spp./*Trichuris* spp. among golden jackals | **p7** |
| **Table S6.** Model predicting infection intensity with *Eucoleus* spp./*Trichuris* spp. among golden jackals | **p7** |
| **Table S7.** Total parasite frequencies (%) and helminth overlap for different canids | **p8** |
| **Figure S1.** Helminth prevalence in scats of foxes, jackals, rural dogs and all mammalian predators | **p9** |
| **Figure S2.** Helminth mono- and coinfections among infected scats of foxes, jackals, rural dogs and all mammalian predators | **p9** |
| **Figure S3.** Proportion of different helminth coinfection categories among coinfected scats of foxes, jackals, rural dogs and all predators | **p10** |
| **Figure S4.** Prevalence of various helminth coinfections among all coinfected predators | **p10** |

**Table S1.** Models predicting coinfection prevalence among predators. Only models with the highest Akaike weight (ΔAICc<2) are described. Statistically significant results are marked in bold.

| Models | Factors | Statistics | Weight | Sum of weights (RVI) |
| --- | --- | --- | --- | --- |
|  |  |  |  |  |
| 1. RODENT | RO | **β_RODENT_=0.9, SE=0.3, p=0.0002** | 0.22 | 1 |
|  |  |  |  |  |
| 2. PLANT+RODENT | P | β_PLANT_=0.3, SE=0.3, p=0.3 | 0.13 | 0.37 |
|  |  |  |  |  |
|  | RO | **β_RODENT_=0.9, SE=0.3, p=0.0001** |  | 1 |
|  |  |  |  |  |
| 3. RODENT+FISH | RO | **β_RODENT_=0.9, SE=0.2, p=0.0001** | 0.11 | 1 |
|  |  |  |  |  |
|  | F | β_FISH_=-0.7, SE=0.9, p=0.4 |  | 0.33 |
|  |  |  |  |  |
| 4. RODENT+GAME | RO | **β_RODENT_=0.9, SE=0.3, p=0.0003** | 0.09 | 1 |
|  |  |  |  |  |
|  | G | β_GAME_=-0.2, SE=0.3, p=0.6 |  | 0.29 |
|  |  |  |  |  |

**Table S2.** Models predicting infection prevalence with *Eucoleus* spp*./Trichuris* spp. among predators. Only models with ΔAICc<2 are described. Statistically significant results are marked in bold.

| Models | Factors | Statistics | Weight | Sum of weights (RVI) |
| --- | --- | --- | --- | --- |
|  |  |  |  |  |
| 1. REPTILE+RODENT | REP | **β_REPTILE_=1.7, SE=0.7, p=0.006** | 0.25 | 0.98 |
|  |  |  |  |  |
|  | RO | **β_RODENT_=1.1, SE=0.3, p=0.00002** |  | 1 |
|  |  |  |  |  |
| 2. BIRD+REPTILE+RODENT | B | β_BIRD_=0.3, SE=0.2, p=0.18 | 0.22 | 0.45 |
|  |  |  |  |  |
|  | REP | **β_REPTILE_=1.9, SE=0.7, p=0.005** |  | 0.98 |
|  |  |  |  |  |
|  | RO | **β_RODENT_=1.1, SE=0.3, p=0.00001** |  | 1 |
|  |  |  |  |  |
| 3. GAME+REPTILE+RODENT | G | β_GAME_=-0.3, SE= 0.3, p=0.35 | 0.14 | 0.34 |
|  |  |  |  |  |
|  | REP | **β_REPTILE_=1.7, SE=0.7, p=0.007** |  | 0.98 |
|  |  |  |  |  |
|  | RO | **β_RODENT_=1.0, SE=0.3, p=0.00007** |  | 1 |
|  |  |  |  |  |
| 4. BIRD+GAME+REPTILE+RODENT | B | β_BIRD_=0.3, SE=0.3, p=0.2 | 0.10 | 0.45 |
|  |  |  |  |  |
|  | G | β_GAME_=-0.2, SE=0.3, p=0.5 |  | 0.34 |
|  |  |  |  |  |
|  | REP | **β_REPTILE_=1.8, SE=0.7, p=0.005** |  | 0.98 |
|  |  |  |  |  |
|  | RO | **β_RODENT_=1.1, SE=0.3, p=0.00005** |  | 1 |
|  |  |  |  |  |
| 5. PLANT+REPTILE+RODENT | P | β_PLANT_=-0.1, SE= 0.3, p=0.8 | 0.09 | 0.28 |
|  |  |  |  |  |
|  | REP | **β_REPTILE_=1.8, SE=0.7, p=0.006** |  | 0.98 |
|  |  |  |  |  |
|  | RO | **β_RODENT_=1.1, SE=0.2, p=0.00002** |  | 1 |
|  |  |  |  |  |

**Table S3.** Models predicting infection intensity with *Eucoleus* spp./*Trichuris* spp. among predators. Only models with ΔAICc<2 are described. Statistically significant results are marked in bold.

| Models | Factors | Statistics | Weight | Sum of weights (RVI) |
| --- | --- | --- | --- | --- |
|  |  |  |  |  |
| 1. GAME | G | **β_GAME_=0.6, SE= 0.3, p=0.048** | 0.12 | 0.82 |
|  |  |  |  |  |
| 2. GAME+REPTILE | G | **β_GAME_=0.7, SE= 0.3, p=0.028** | 0.10 | 0.82 |
|  |  |  |  |  |
|  | REP | β_REPTILE_=0.7, SE=0.5, p=0.2 |  | 0.43 |
|  |  |  |  |  |
| 3. GAME+BIRD | G | β_GAME_=0.6, SE= 0.3, p=0.05 | 0.09 | 0.82 |
|  |  |  |  |  |
|  | B | β_BIRD_=-0.3, SE=0.3, p=0.2 |  | 0.43 |
|  |  |  |  |  |

**Table S4.** Models predicting infection prevalence with *Toxocara canis* among red foxes. Only models with ΔAICc<2 are described. Statistically significant results are marked in bold.

| Models | Factors | Statistics | Weight | Sum of weights (RVI) |
| --- | --- | --- | --- | --- |
|  |  |  |  |  |
| 1. PLANT+GAME | P | **β_PLANT_=1.4, SE=0.6, p=0.017** | 0.16 | 0.84 |
|  |  |  |  |  |
|  | G | β_GAME_=0.9, SE=0.6, p=0.1 |  | 0.53 |
|  |  |  |  |  |
| 2. INSECT | I | β_INSECT_=-0.6, SE= 0.8, p=0.4 | 0.14 | 0.33 |
|  |  |  |  |  |
| 3. PLANT+INSECT | P | **β_PLANT_=1.4, SE=0.6, p=0.02** | 0.07 | 0.84 |
|  |  |  |  |  |
|  | I | β_INSECT_=-0.6, SE= 0.8, p=0.4 |  | 0.33 |
|  |  |  |  |  |
| 4. PLANT+GAME+BIRD | P | **β_PLANT_=1.4, SE=0.6, p=0.018** | 0.07 | 0.84 |
|  |  |  |  |  |
|  | G | β_GAME_=0.9, SE=0.6, p=0.1 |  | 0.53 |
|  |  |  |  |  |
|  | B | β_BIRD_=0.4, SE=0.5, p=0.5 |  | 0.32 |
|  |  |  |  |  |
| 5. PLANT+GAME+INSECT | P | **β_PLANT_=1.4, SE=0.6, p=0.017** | 0.07 | 0.84 |
|  |  |  |  |  |
|  | G | β_GAME_=0.9, SE=0.6, p=0.1 |  | 0.53 |
|  |  |  |  |  |
|  | I | β_INSECT_=-0.5, SE=0.8, p=0.5 |  | 0.33 |
|  |  |  |  |  |
| 6. PLANT+GAME+RODENT | P | **β_PLANT_=1.5, SE=0.5, p=0.016** | 0.06 | 0.84 |
|  |  |  |  |  |
|  | G | β_GAME_=1.0, SE=0.6, p=0.1 |  | 0.53 |
|  |  |  |  |  |
|  | RO | β_RODENT_=0.2, SE=0.6, p=0.7 |  | 0.27 |
|  |  |  |  |  |
| 7. BIRD+RODENT | B | β_BIRD_=0.3, SE=0.5, p=0.5 | 0.06 | 0.32 |
|  |  |  |  |  |
|  | RO | β_RODENT_=-0.1, SE=0.5, p=0.8 |  | 0.27 |
|  |  |  |  |  |

**Table S5.** Models predicting infection prevalence with *Eucoleus* spp./*Trichuris* spp. among golden jackals. Only models with the highest Akaike weight (ΔAICc<2) are described. Statistically significant results are marked in bold.

| Models | Factors | Statistics | Weight | SUM OF WEIGHTS (RVI) |
| --- | --- | --- | --- | --- |
|  |  |  |  |  |
| 1. RODENT | RO | **β_RODENT_=2.6, SE=0.7, p=0.0002** | 0.43 | 1 |
|  |  |  |  |  |
| 2. RODENT+GAME | G | β_GAME_=0.2, SE=0.8, p=0.8 | 0.32 | 0.25 |
|  |  |  |  |  |
|  | RO | **β_RODENT_=2.6, SE=0.8, p=0.0007** |  | 1 |
|  |  |  |  |  |

**Table S6.** Model predicting infection intensity with *Eucoleus* spp./*Trichuris* spp. among golden jackals. Only models with the highest Akaike weight (ΔAICc<2) are described. Statistically significant results are marked in bold.

| Model | Factors | Statistics | Weight | SUM OF WEIGHTS (RVI) |
| --- | --- | --- | --- | --- |
|  |  |  |  |  |
| 1. GAME+RODENT | G | **β_GAME_=2.6, SE=1.2, p=0.03** | 0.56 | 0.75 |
|  |  |  |  |  |
|  | RO | **β_RODENT_=4.1, SE=1.3, p=0.0008** |  | 0.97 |
|  |  |  |  |  |

**Table S7.**

Total parasite frequencies (%) and helminth overlap for different canids. Euc/Tri: *Eucoleus* spp.*/Trichuris* spp. Data for urban and rural dogs are from Tull et al. 2020 and Tull et al. 2022, respectively.

| Parasite | urban dog vs rural dog | | urban dog vs red fox | | urban dog vs jackal | | rural dog vs red fox | | rural dog vs jackal | | jackal vs red fox | |
| --- | --- | --- | --- | --- | --- | --- | --- | --- | --- | --- | --- | --- |
| Taeniidae | 3 | 76 | 3 | 100 | 3 | 57 | 76 | 100 | 76 | 57 | 57 | 100 |
| *Euc/Tri* | 2 | 18 | 2 | 105 | 2 | 14 | 18 | 105 | 18 | 14 | 14 | 105 |
| *Toxocara canis* | 22 | 5 | 22 | 18 | 22 | 3 | 5 | 18 | 5 | 3 | 3 | 18 |
| *Toxascaris leonina* | 0 | 0 | 0 | 1 | 0 | 3 | 0 | 1 | 0 | 3 | 3 | 1 |
| *Uncinaria stenocephala* | 23 | 17 | 23 | 24 | 23 | 18 | 17 | 24 | 17 | 18 | 18 | 24 |
| Total | 50 | 116 | 50 | 248 | 50 | 95 | 116 | 248 | 116 | 95 | 116 | 95 |
| Helminth overlap | 29.8% | | 30.7% | | 34.4% | | 84.2% | | 99.6% | | 84.1% | |

**References**

Tull, A., Moks, E., Laurimaa, L., Keis, M., Süld, K., 2020. Endoparasite infection hotspots in Estonian urban areas. J. Helminthol. 94, e104, 1–9. <https://doi.org/10.1017/S0022149X19000920>

Tull, A., Valdmann, H., Rannap, R., Kaasiku, T., Tammeleht, E., Saarma, U., 2022. Free-ranging rural dogs are highly infected with helminths, contaminating environment nine times more than urban dogs. J. Helminthol. 96, e19. <https://doi.org/10.1017/S0022149X22000116>

**
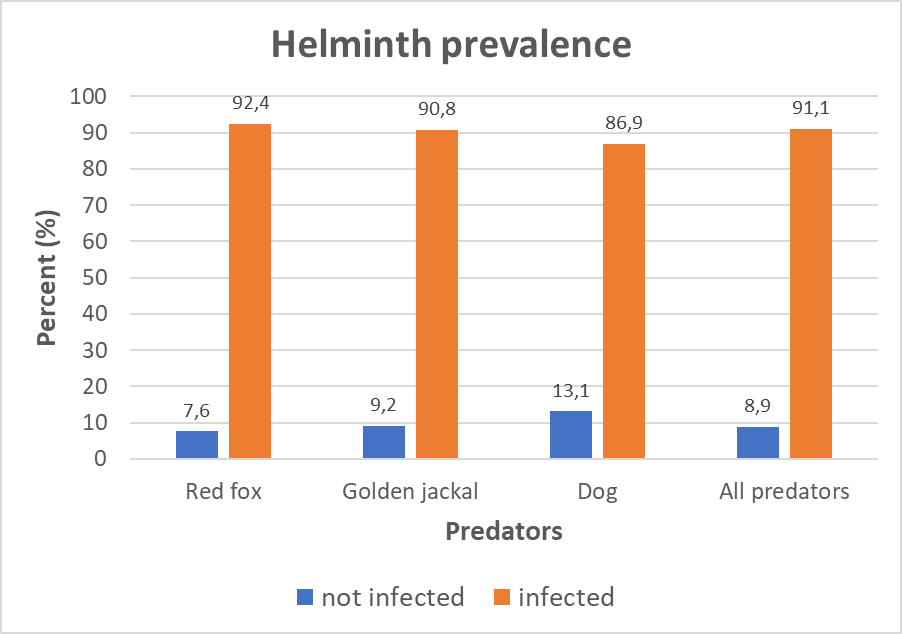
**

**Fig. S1.** Helminth prevalence in scats of red foxes (n = 131), golden jackals (n = 65), rural dogs (n = 84) and all mammalian predators (n = 315).

**
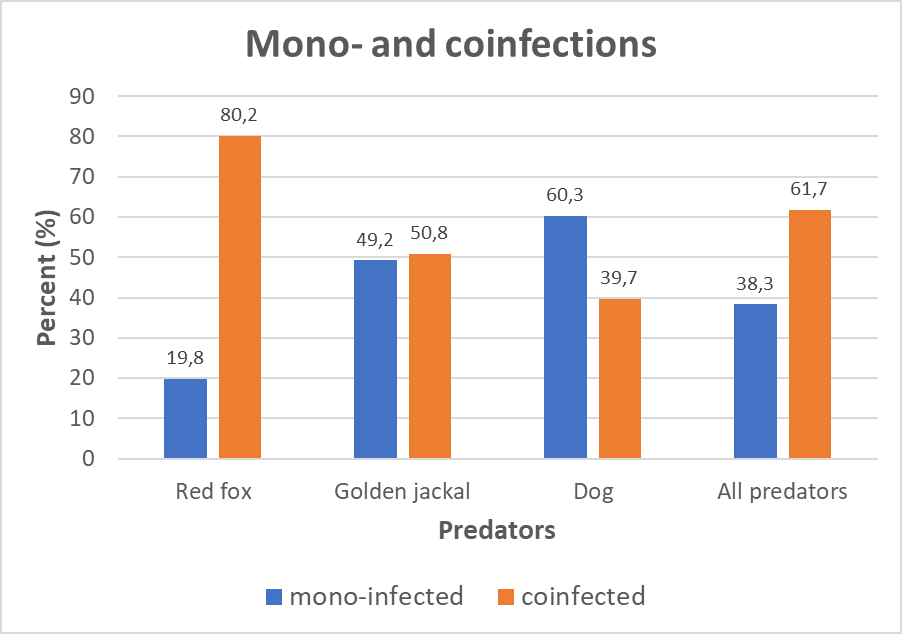
**

**Fig. S2.** Helminth mono- and coinfections among infected scats of red foxes (n = 121), golden jackals (n = 59), rural dogs (n = 73) and all mammalian predators (n = 287).

**
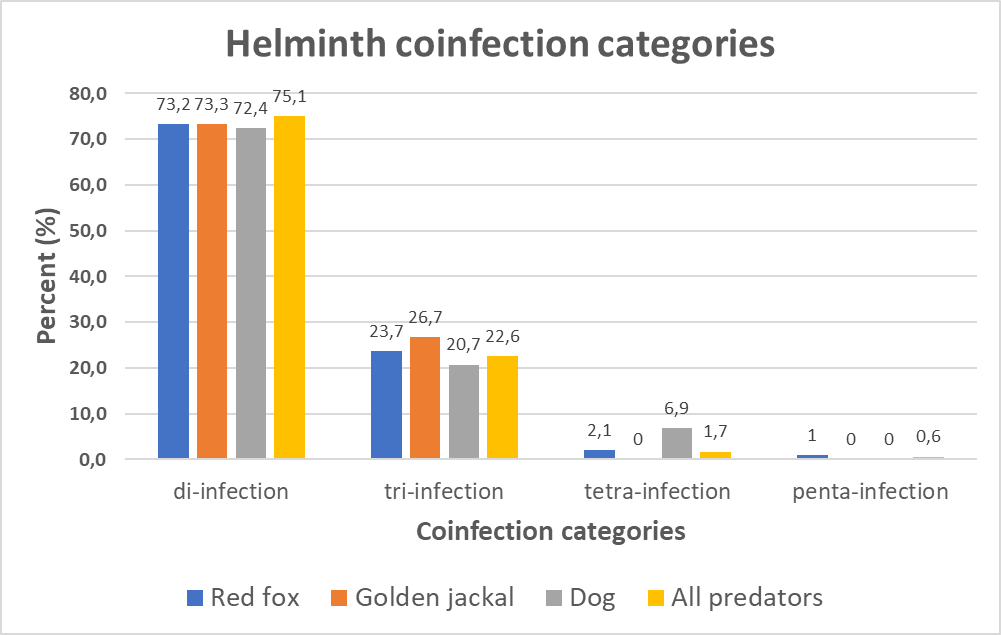
**

**Fig. S3.** Proportion of different helminth coinfection categories among coinfected scats of red foxes (n = 97), golden jackals (n = 30), rural dogs (n = 29) and all predators (n = 177).

**
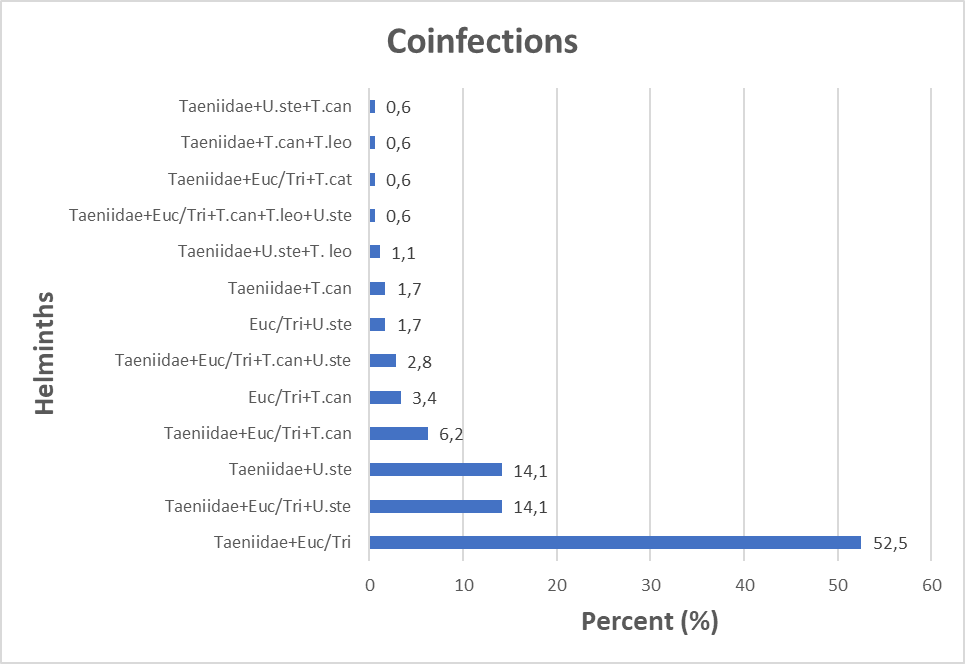
**

**Fig. S4.** Prevalence of various helminth coinfections among all coinfected predators (n = 177). Euc/Tri: *Eucoleus* spp.*/Trichuris* spp; T.can: *Toxocara canis*; T.cat: *Toxocara cati*; T.leo: *Toxascaris leonina*; U. ste: *Uncinaria stenocephala.*
